# Advancing suicide prevention in Germany, Austria and Switzerland: a qualitative study

**DOI:** 10.3389/fpubh.2024.1378481

**Published:** 2024-05-30

**Authors:** Sophia Werdin, Kaspar Wyss

**Affiliations:** ^1^Swiss Tropical and Public Health Institute, Allschwil, Switzerland; ^2^University of Basel, Basel, Switzerland

**Keywords:** mental health, suicide prevention, national prevention strategies, prevention programs, multi-level prevention, collaboration, lived experience, qualitative research

## Abstract

**Introduction:**

Suicide is a significant public health problem, impacting individuals, families and communities worldwide. Effective suicide prevention requires a comprehensive approach with diverse integrated interventions and collaboration across sectors, stakeholders and professions. This study aims to identify challenges, gaps and success factors in current suicide prevention efforts in Germany, Austria and Switzerland, providing specific recommendations for advancement.

**Methods:**

We conducted online, semi-structured interviews with 36 suicide prevention experts from Germany, Austria and Switzerland, incorporating perspectives from policy, science and practice. Interviews were conducted between September 2022 and February 2023, audio-recorded, transcribed verbatim and analyzed using the Framework method.

**Results:**

Despite progress in national strategies and coordinated efforts for suicide prevention, challenges such as resource scarcity, stigma and structural issues in psychiatric and psychotherapeutic care persist. The interviewees identified several areas for advancement, including developing targeted prevention measures for men and older people, strengthening collaboration across sectors, stakeholders and professions, and increasing the involvement of individuals with lived experience. While the COVID-19 pandemic has exacerbated challenges in psychiatric and psychotherapeutic care, it has concurrently strengthened interest in suicide prevention among policymakers and the media.

**Discussion:**

National suicide prevention strategies play a crucial role in setting priorities, raising public awareness, and guiding action. However, since most suicide prevention efforts are still predominantly health sector-driven, a more comprehensive approach is needed to promote the involvement of all relevant actors and address suicidality as a collective societal responsibility. Tailoring prevention programs for risk groups like older people and men is important, as these populations show high suicide rates and face a lack of targeted interventions. Our study underscores the importance to continuously monitor, refine and strengthen collaborative and evidence-based suicide prevention efforts.

## 1 Introduction

Suicide is a global public health problem, impacting individuals, families and communities around the globe (1). Each year, about 700,000 people die by suicide worldwide, with the number of suicide attempts and individuals suffering from suicidal thoughts being substantially higher (1). In 2021, epidemiological data indicated an average suicide rate of 10.2 per 100,000 population in the European Union (2). Notably, Germany (10.3 per 100,000), Austria (11.9 per 100,000), and Switzerland (11.5 per 100,000) recorded suicide rates surpassing the European average (2).

The reasons for suicidality are complex, involving multi-level interactions among various risk factors (3). Suicidal thoughts are often caused by mental illness, particularly major depressive disorder and bipolar disorder, underscoring the importance of their effective treatment (4). Suicide prevention (SP) requires a coordinated, sustainable approach that integrates diverse measures and multi-level collaboration across sectors, stakeholders and professions (5). No single measure alone is sufficient to prevent suicides (3). The 4-level-intervention concept by the European Alliance against Depression (6) exemplifies a comprehensive, widely implemented and evaluated community-based program designed to improve care for patients with depression and prevent suicidal behavior (7). The concept incorporates 1) interventions for primary and mental health care professionals, 2) interventions for the general public and community, 3) interventions for community facilitators, gatekeepers and stakeholders, and 4) interventions for patients, high-risk groups and their relatives (7).

The range of SP measures is broad. Population-based interventions that can contribute to preventing suicides include, for example, restricting access to suicidal means, suicide preventive media coverage and public awareness campaigns (8). High-risk approaches include SP interventions for specific risk groups, such as prisoners, older, isolated individuals and drug or alcohol dependents as well as improved diagnosis and treatment of psychiatric disorders (8). Understanding the reasons for suicide risk and the barriers to accessing mental health support is crucial to the design of targeted interventions (9, 10). In order to ensure that actual needs are met, the involvement of individuals with suicidal experience and their relatives in the design, implementation and evaluation of SP initiatives is considered invaluable (11, 12).

Over recent decades, the World Health Organization (WHO) has raised global awareness of suicide, leading to the development of comprehensive SP strategies in many countries (5). A national SP strategy is a government-led, nationwide initiative that systematically addresses suicidal behavior through diverse approaches and integrated, countrywide measures (5, 13). While often having similar components, such as public education, crisis intervention and suicide monitoring (3, 5), national strategies may differ, for example, with respect to the level of community involvement or participating agencies (14). According to the assessment of the WHO in 2017, almost 10% of low- and lower middle-income countries and about one-third of upper middle- and high-income countries have implemented a stand-alone government-adopted national SP strategy (15).

In the Central European countries of Germany, Austria and Switzerland, various associations, organizations, foundations and networks are committed to SP at the national or regional level, connecting expertise and political competence from various professions and enhancing collaboration and research in SP. Each of these countries has established distinct national SP initiatives to guide SP efforts. In Germany, the National Suicide Prevention Program (NaSPro) contributes as a nationwide specialist network for exchange and knowledge transfer on suicidality and SP (16). In 2021, a report on the current state and prospects of SP in Germany was compiled by the expertise of NaSPro (17). In May 2024, the suicide prevention strategy for Germany was introduced, comprising measures and recommendations in three overarching areas of action (18). Other organizations that contribute to SP in Germany are, for example, the German Depression Foundation and the German Association for Suicide Prevention.

The Austrian SP strategy, Suicide Prevention Austria (SUPRA), features strategic and operational objectives for national SP along with subordinate measures, including corresponding target values and responsibilities (19). An interim report on the implementation of SUPRA is published annually. In the same year of SUPRA’s implementation, an SP coordination office was established at the national public health institute with the responsibilities of implementing the measures proposed by SUPRA, consolidating ongoing SP efforts in the federal states, establishing high-quality national reporting on suicide and coordinating cross-sectoral collaboration (20). Other organizations that contribute to SP in Austria are, for example, the Austrian Association for Suicide Prevention and Pro Mente Austria.

The Swiss national action plan for suicide prevention, developed by the Swiss Federal Office of Public Health, the Swiss Conference of Cantonal Directors of Public Health and the foundation Health Promotion Switzerland, comprises 10 goals with 19 specific measures to advance SP in Switzerland (21). An interim status report on its implementation was published in 2021 (22). Other organizations that contribute to SP in Switzerland are, for example, the Initiative for the Prevention of Suicide in Switzerland and Pro Mente Sana. National efforts in these three countries are complemented by international SP organizations, such as the International Association for Suicide Prevention and the European Alliance against Depression.

In scientific publications, government reports and practice, terms such as *strategy*, *program, action plan* and *project* are not consistently used. To ensure clarity, we use the term *strategy* to refer to national concepts (including national action plan) setting broad goals and direction, the term *program* for long-term, comprehensive efforts aligned with the overarching strategy and encompassing a range of interconnected projects and the term *project* for temporary initiatives targeting specific objectives within a program.

In Germany, Austria and Switzerland, three neighboring countries in Central Europe with similar socio-cultural, economic and political-organizational characteristics, suicide rates have been a topic of concern for several decades. Although these nations boast high standards of living and advanced health care systems, suicidality continues to burden society. This study aims to identify the challenges, gaps and success factors in current SP efforts in Germany, Austria and Switzerland in order to derive specific recommendations for advancing SP in these countries.

## 2 Methods

A qualitative study design was chosen to provide in-depth, differentiated descriptions of expert knowledge, experiences and opinions relevant to the research interest.

### 2.1 Study setting

Our study focuses on the SP landscape in Germany, Austria and Switzerland, three high-income and politically stable countries in Central Europe. In these countries, the planning and decision-making in SP predominantly reside at the level of federal states or cantons. In Germany and Austria, the federal states, as well as the cantons in Switzerland, are subnational entities with their own governments and parliaments, ensuring regional autonomy through decentralized administration within the national framework.

From September 2022 to February 2023, we conducted one-on-one online interviews with SP experts from Germany, Austria and Switzerland to investigate SP measures and strategies employed in these countries.

### 2.2 Study participants and recruitment

In total, 36 SP experts participated in the study. They are referred to as experts due to their specific knowledge, relevant experience and roles as informants (23). In this study, we were interested in expert knowledge based on professional involvement in SP. Individuals were considered experts if they engaged in SP as part of their professional duties, such as planning, coordinating, implementing or evaluating SP measures. The identification and selection of experts was based on the judgment of the researchers. The first author (SW) screened relevant organizations for potential participants and discussed this approach and the selected individuals with the co-author (KW) and other colleagues working in the field of SP research.

12 experts from each of the three countries – Germany, Austria and Switzerland – were interviewed. The participants were categorized according to their primary engagement in policy, science or practice. To ensure equal representation of the different perspectives, 12 experts from each of the three domains were included. Experts with a policy background include, for example, employees of federal health agencies and members of international, national or regional SP societies, associations, foundations and competence groups. Most scientific experts are researchers at universities or research institutes of university hospitals working in suicide and SP research. Experts from practice include, for example, psychiatrists, psychotherapists, employees from counseling services and others who regularly interact directly with individuals at suicide risk.

Many of the 36 experts involved in our study, despite having a primary affiliation with one of the three domains – policy, science or practice – are actively engaged in several roles across the broader field of SP. For instance, some experts primarily categorized under the policy domain also contribute to scientific research. Similarly, several researchers engage in policy-making processes, and many clinical health professionals conduct scientific studies in suicide and SP research in addition to providing clinical care. The categorization of the interviewees into professional domains is intended not as a strict demarcation but merely to ensure that the different perspectives are equally incorporated.

Study participants were selected through purposive sampling and were approached via e-mail. Of the 68 SP experts approached, 16 did not respond and 16 declined to participate, mostly due to time constraints. No expert dropped out after agreeing to participate in the study.

### 2.3 Data collection

Online, semi-structured interviews were conducted via Zoom, guided by an interview protocol with open-ended questions (see Supplementary material 1). The instrument was pretested for logic, comprehensibility and completeness with two non-experts. The following topics were covered by the interview guide: 1) aspects on the national SP approach, 2) the evaluation and 3) effectiveness of SP measures, 4) the availability and quality of data and evidence in SP, 5) challenges in SP, 6) the impact of the coronavirus disease 2019 (COVID-19) pandemic and 7) best practice elements and optimization potentials. The emphasis on certain interview topics aligned with the experts’ primary perspective. Study participants received the guide via e-mail one week prior to the interview.

The first author (SW, female, Ph.D. candidate at the Swiss Tropical and Public Health Institute, trained and experienced in qualitative research methods) interviewed all experts. Except for the previous collaboration with three Swiss experts from the sample, the interviewer had no contact with the other interviewees prior to this study. To ensure reflexivity, SW regularly engaged in self-reflection to scrutinize personal assumptions and preconceptions about the research topic. In conducting numerous interviews on the same topics with identical questions that repeatedly elicited similar responses from interviewees, assumptions began to form regarding future interviews. To counteract interviewer bias, careful attention was given to ensure that questions in subsequent interviews were posed openly and objectively, without revealing any pre-existing assumptions. The identity of the researcher, namely her female gender, young age and status as a doctoral student, may have influenced the willingness to participate or the interview process. To reduce any possible influence, we used standardized scripts for the recruitment process and practiced neutral questioning. Experiences, thoughts, evolving insights and reflections were documented, discussed with the co-author (KW) and other colleagues, and consulted during the data analysis. The choice of research methodology was influenced by the research objectives and the professional experience of the interviewer.

All interviews were conducted in German (with the exception of one interview in English), audio-recorded using an encrypted voice recorder and transcribed verbatim following the basic transcription system of Dresing and Pehl (24). During transcription, all names and other details that would allow a direct link to an individual were anonymized. Upon request, study participants were provided with a copy of the anonymized transcript of their interview.

### 2.4 Data analysis

Data were analyzed using the Framework method for a systematic approach to managing and mapping qualitative data (25). Thematic categories (codes) were deductively derived from the interview guide. These codes were systematically applied to the data using a coding tree, which served as a structured framework for organizing and analyzing qualitative data. The framework facilitated the identification and retrieval of relevant text segments and enabled a systematic examination of the data based on the main themes and associated sub-themes. To ensure consistency, the interviewer, who had good data understanding from conducting and transcribing the interviews, also coded and analyzed the data. Data reduction and analysis was based on a theme matrix “cases x codes”, which allowed data comparison across cases as well as within a single case (25). Data management and analysis were conducted using MAXQDA.

This manuscript presents our findings on the perceived role of SP and national SP strategies in the countries of interest, conditions for collaboration in SP, the acceptance of SP measures as well as the impact of the COVID-19 pandemic on SP, along with associated challenges, best practices and areas for enhancement. A forthcoming, complementary manuscript will discuss expert experiences and opinions on the evaluation of SP measures and the availability and quality of suicide data, along with associated challenges, best practices and areas for enhancement.

### 2.5 Ethics statement

Participation was voluntary. All study participants received detailed study information via mail prior to data collection, which explained the objectives and procedures of the research project. The interviews were based on written, informed consent. There was no compensation for study participation. This study, reviewed by the Ethics Committee of Northwestern and Central Switzerland (ID: Req-2022-00881) in August 2022, was determined not to require ethical approval. Reporting was guided by the Consolidated Criteria for Reporting Qualitative Research: 32-item checklist (26).

## 3 Results

### 3.1 Sample characteristics

**Figure 1:**
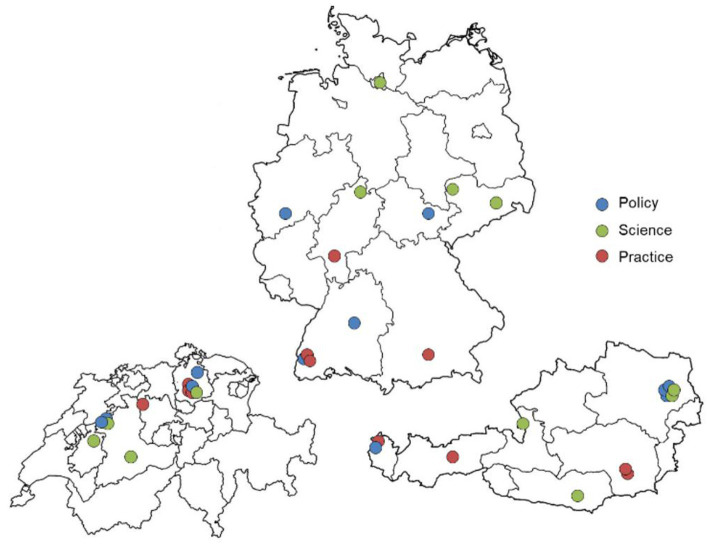
Geographic distribution of study participants with their main professional perspective (adjusted country size), own illustration.

The study comprised a sample of 36 experts, consisting of 15 females (41.7%) and 21 males with an average age of 53 years, both in the total sample and within each professional perspective (see Table 1). Interview duration varied from 22 to 69 minutes, averaging at 42.5 minutes. Study participants were spread across 17 different federal states or cantons in Germany, Austria and Switzerland (see Figure 1).

**Table 1:** Demographic characteristics of study participants and duration of interviews (*N* = 36).

Characteristics	Policy (*n* = 12)	Science (*n* = 12)	Practice (*n* = 12)
Gender [*n*]			
Female	4	6	5
Male	8	6	7
Age [*years*]			
Mean (*SD*)	53 (8.6)	53 (9.1)	53 (10.7)
Range	38–67	43–76	36–67
Country of employment [*n*]			
Switzerland	4	4	4
Germany	4	4	4
Austria	4	4	4
Duration of the interviews [*min*]			
Mean (*SD*)	42.5 (10.4)		
Range	22.2–68.9		

The duration of professional SP experience ranged from 3 to 45 years, averaging at 19.5 years. 77.8% (*n* = 28) of experts have been professionally engaged in SP for more than 10 years. The proportion of their working time devoted to SP varied from 5 to 100%. Study participants noted difficulties in accurately assessing this percentage due to fluctuating workloads depending on SP projects and events. Additionally, the integration of SP within broader mental health services, particularly in hospital settings, made distinct demarcation challenging. Moreover, a substantial engagement in SP is based on voluntary work, with many experts dedicating a significant portion of their free time to related activities.

### 3.2 Qualitative findings

The following sections summarize expert statements on the perceived relevance of SP in their country, related cross-sectoral, cross-regional and interprofessional collaboration, the acceptance of SP services and projects, further challenges, gaps and potentials for advancement in current SP efforts and the impact of the COVID-19 pandemic on SP. The main findings from our study, based on the statements of participating experts, are summarized in Table 2.

**Table 2:** Summary of the main findings based on the statements of the participating experts.

Topic	Main findings
Perceived relevance of suicide prevention	The perceived significance of suicide prevention has grown in recent years, accompanied by an increase in related efforts and awareness.
	National suicide prevention strategies hold an important role, providing a concerted framework for action and underlining political commitment to suicide prevention.
	Suicide prevention and related research are perceived as underfunded compared to other public health areas.
	Stigma and misconceptions around suicidality persist, requiring comprehensive public awareness campaigns.
	In Germany, Austria and Switzerland, engagement and resource allocation for suicide prevention vary significantly across federal states or cantons.
Collaboration in suicide prevention	National strategies, networks, initiatives and organizations for suicide prevention foster collaboration, networking and learning.
	Personal contacts and awareness among relevant stakeholders and political decision-makers are crucial for effective collaboration.
	Decentralized regulatory authority, stigmatization of suicidality, lack of awareness and insufficient resources hamper collaboration in suicide prevention.
	In Switzerland, language barriers between cantons with different national languages hamper cross-regional collaboration.
	There is a need for stronger collaboration and networking across sectors, stakeholders and professionals.
Acceptance of suicide prevention services and projects	The accessibility of suicide prevention services largely influences their acceptance and utilization by the target group.
	Interventions need to be tailored to meet the target group’s specific needs and characteristics.
	Incorporating the perspectives of individuals with lived experience and their relatives in suicide prevention measures enhances their relevance, feasibility and effectiveness. In practice, however, the involvement of these perspectives is often limited.
Further challenges, gaps and potentials for advancement in suicide prevention efforts	There is a need to strengthen psychiatric and psychotherapeutic care, including suicide risk assessment and care for individuals after a suicide attempt, as well as support for relatives after a suicide.
	Limited availability and accessibility of mental health and psychosocial care in rural areas can contribute to higher suicide rates.
	Targeted suicide prevention measures are needed for men and older adults, who experience high suicide rates.
	Experts from Germany and Austria advocate for the establishment of a national 24/7 telephone hotline that offers support in crisis situations.
Impact of the COVID-19 pandemic on suicide prevention	Experts in all three countries reported a surge in demand for mental health support during the COVID-19 pandemic, particularly among children and adolescents.
	The COVID-19 pandemic exacerbated existing structural challenges in mental health care, such as long waiting times and insufficient treatment resources.
	The COVID-19 pandemic increased awareness of mental health and suicidality among the public, the media and policymakers.

#### 3.2.1 Perceived relevance of suicide prevention

Most experts from all three countries and professional backgrounds noted that SP in general tends to be a rather low priority in public health compared to other health issues. However, they also pointed out that its significance has grown noticeably in recent years. This positive trend can be exemplified by initiatives such as the substantial funding by the foundation Health Promotion Switzerland of four new suicide prevention projects in Switzerland since 2021. The increase in awareness among society and policymakers is accompanied by the development from sporadic, urban-centric SP measures to more comprehensive and coordinated efforts. According to the study participants, national SP strategies have played a pivotal role in this context, providing a structured framework, demonstrating political commitment, fostering collaboration and encouraging all federal states or cantons to address SP.

“There are two success factors that are important for us. One of them is that the suicide prevention program is an official program of the Austrian Ministry of Health, which […] somehow gives it much higher importance, and in a way, gently encourages, to put it carefully, the individual federal states to participate. […] The second [success factor] is the setting. That there is this coordination office […] and that it is located at the Austrian Public Health Institute results in so many synergy effects.” (Participant 5, male, policy, Austria)

Experts from Germany and Austria noted that recent debates on assisted suicide legislation have enhanced the prominence and recognition of SP in public and political spheres.

“This year in Germany, we are talking a lot about assisted suicide and its legal regulation. Whenever there is some political occasion – now in November, the legislation will be discussed again – then it [suicide prevention] has a relatively high significance. On occasions like the World Suicide Prevention Day, it has a relatively high relevance. In the general discussion, however, it has rather little prominence.” (Participant 14, female, practice, Germany)

Despite advancements and individual funding measures, many experts from all three countries mentioned the inadequacy of financial investments in SP and related research. The comparatively small number of engaged professionals and the substantial reliance on voluntary work, particularly in policy, underscore the need for more human and financial resources. Many experts indicated that increasing resources for SP and associated research are key opportunities for advancement.

“When you compare the investment in road accident prevention and how many deaths occur by road accidents – it is about as frequent as suicides [in adolescents]. […] Ten to fifteen times more money is invested in road accident prevention than in suicide prevention. This is actually a disgrace. The same applies for cancer research, where the factor is even higher. When it is about research, when it is about understanding how a young person becomes suicidal, it is grotesque how much money is spent on rare diseases in research and how little money is allocated to youth suicide [prevention].” (Participant 36, male, practice, Switzerland)

According to several experts, persistent stigma and misconceptions around suicidality and mental health continue to pose challenges for preventing suicides. Resistance to SP efforts, for example in educational, criminal justice and community settings, indicates a need for more comprehensive public awareness and engagement campaigns.

“There are still instances where we encounter resistance with the topic. […] When we advertise an event with the title ‘Suicide Prevention – Knowledge Helps,’ almost no one comes. But if we rephrase it as ‘Resilience – Strong for Everyday Life,’ we have many participants. This means that it is still the best not to address the topic of suicide and suicidality directly. […] There is still a devaluation, an association with weakness. And those of us who address it [suicide prevention] are sometimes seen as missionaries, trying to convert or help everyone. ‘This is not possible. Because people who want to take their own life will do it anyway.’ We do encounter resistance from time to time, LESS now, but still.” (Participant 30, female, practice, Austria)

According to many experts from all three countries, regional SP commitment differs. The federal structures of Germany, Austria and Switzerland result in varying levels of SP engagement and resource allocation across federal states or cantons as well as urban and rural areas. In these countries, most regulatory authority in the area of public health is at decentralized level, while responsibilities at national level are limited.

“The federal government's hands are tied and the constitution does not allow it to take action in this area. […] Unlike other countries, the federal government in Switzerland primarily has a coordinating role [in suicide prevention]. And this is the major dilemma in Switzerland, especially for the smaller cantons, as they often struggle due to their limited resources.” (Participant 33, male, practice, Switzerland)

#### 3.2.2 Collaboration in suicide prevention

A comprehensive SP approach requires several levels of collaboration. This section first summarizes the experts’ experiences in cross-sectoral collaboration followed by reflections on cross-regional collaboration and interprofessional collaboration in mental health care.

##### 3.2.2.1 Cross-sectoral collaboration

In the past, cross-sectoral collaboration in SP was rather fragmented and sporadic but many experts from Germany, Austria and Switzerland noted a shift toward more systematic interconnectedness. The study participants primarily attributed this improvement to the implementation of national SP strategies and the involvement of various stakeholders in SP societies, associations and other initiatives. Besides health care, collaboration with sectors such as education, media and criminal justice as well as social services is key. Personal contacts and awareness among relevant stakeholders and political decision-makers were deemed crucial for cross-sectoral collaboration.

Experts from several regions, like Carinthia in Austria or Zurich in Switzerland, showcased successful sector and stakeholder collaboration at the regional level.

“The cooperation in Carinthia has actually become very good since we founded SUPRA in 2018/19. The stakeholders involved are the executive branch because they are often confronted with suicides, the Red Cross with the crisis intervention team […] The department of psychiatry in Klagenfurt is very intensively involved, as is the department in Villach. We work very closely with the media, talking about reporting guidelines.” (Participant 20, male, science, Austria)

“What we always try to do is to conduct these so-called suicide reports in Zurich. These are regional exchange forums held over lunch, twice a year. And we try to make them quite accessible. The main goal there is to connect people who are professionally in contact with suicidal individuals. These can be quite diverse, including, for example, the police, hospitals and someone from social work in schools, for instance. We really aim to improve this networking.” (Participant 11, female, policy, Switzerland)

Several experts from all three countries considered collaboration with the media to be comparatively good. In particular, study participants from Austria highlighted strong and long-standing media cooperation, exemplified by initiatives like the Papageno Media Award, an annually awarded media prize for suicide preventive reporting. However, several interviewees from Germany, Austria and Switzerland identified shortcomings and gaps in collaboration, for example with sectors such as education and the criminal justice system.

##### 3.2.2.2 Cross-regional collaboration

The level of cross-regional collaboration between relevant actors was described differently, largely depending on existing networks, initiatives and the commitment of regional stakeholders. Again, many experts considered national SP strategies to be of great value in promoting collaboration across regions. For example, Austrian experts highlighted that SUPRA facilitates nationwide networking and learning, connecting stakeholders across federal states.

“It is good that we have this overarching entity, namely SUPRA, that connects ALL federal states. And we do not have to reinvent the wheel but can really see how such projects as GO-ON [suicide prevention program in Styria] develop in all federal states. And then we can learn from each other, from the experiences of other federal states. And through SUPRA top-level networking takes place.” (Participant 30, female, practice, Austria)

Similarly, organizations such as the German Society for Suicide Prevention and the working groups of NaSPro were described to foster collaboration and best practice development in Germany.

“The German Society for Suicide Prevention offers a very high-quality exchange during its conferences, in my opinion. But what I would like to emphasize even more are the working groups of NaSPro. They have come together in various areas to ensure that every professional working in the field should actually participate and contribute here [in suicide prevention]. So that we do NOT work in parallel in many places. Instead, we consolidate the expertise and also develop a certain political pressure, professional pressure and work toward best practices. This is actually something that is quite well developed.” (Participant 1, male, policy, Germany)

Some experts from Austria and Switzerland pointed out that cross-regional collaboration and the establishment of personal contacts is comparatively easier in these countries due to their smaller size. In Switzerland, however, challenges in cross-regional collaboration exist due to language barriers between cantons with different national languages.

Apart from cross-regional networks, numerous initiatives and projects run in a rather uncoordinated manner, according to several experts. To avoid parallel structures and unify efforts, some experts from Germany advocated for a national SP coordination office, which would streamline initiatives and foster more cohesive approaches and interventions. Furthermore, enhancing information about available services and projects through means like a national information website was recommended.

“I always notice that there are already a lot of good offers. That is true. It is important to really appreciate that. […] In my opinion, though, it all happens in a very uncoordinated manner. […] And that is also one of the core demands of various associations, including NaSPro, DGS [German Society for Suicide Prevention] and others: we need something like a central coordination office […] We now have several networks in Germany: in Dresden, in Berlin, in Cologne, in Frankfurt, in Thuringia. […] But there is not really a concerted effort among them to develop a common strategy.” (Participant 13, female, science, Germany)

##### 3.2.2.3 Interprofessional collaboration in mental health care

Most experts in our study deemed the interconnectedness and collaboration among mental health care professionals insufficient. Initiatives such as the connection service in Styria (Austria) and so-called bridging conferences in Zurich (Switzerland) illustrate regional efforts to improve the transition from inpatient to outpatient care.

“We are currently in the process of implementing an enhanced connection service, so that people are connected with the counseling services in the region before they are discharged. When they go to the hospital, they are introduced to these services and an initial appointment is scheduled before their discharge. This has been in place for about a year and a half now and it has been very successful. It is better than it has ever been before.” (Participant 32, male, practice, Austria)

Apart from such scattered initiatives, the gap between inpatient and outpatient treatment settings for suicidal individuals remains, according to most study participants.

“There is a gap, I would say. And it does not exist because they [the health care professionals] do not want to collaborate, but often because they lack the time and financial resources. […] Because the inpatient health care providers often find themselves in a situation where they have very little time for a large number of patients. And the outpatient therapists are not compensated for the services they provide when they participate in roundtable discussions or visit someone in a hospital setting. There is a real lack of both time and financial resources, which creates a gap in between.” (Participant 22, female, practice, Switzerland)

Long waiting times for psychotherapeutic outpatient treatment highlight the need for more intensive post-discharge support. In some regions in Germany, Austria and Switzerland, projects like the Attempted Suicide Short Intervention Program (ASSIP) were therefore implemented.

“Psychotherapists have a waiting period of at least three months here in the urban area. Getting a psychotherapy appointment more quickly is rarely possible. I think it is appropriate to assume that the post-discharge care following inpatient treatment for a suicide attempt is not adequate. This was the reason and motivation for us to focus on the ASSIP project […] Now, we are offering it here with hoping that we can contribute to an improvement.” (Participant 28, female, practice, Germany)

Overall, despite advances in SP collaboration, most experts from all three countries highlighted the need for stronger collaboration and networking among professionals, stakeholders and sectors. The hindering factors identified, including decentralized regulatory authority, stigmatization of suicidality, lack of awareness and limited resources, underscore the complexity of collaboration in SP.

#### 3.2.3 Acceptance of suicide prevention services and projects

The majority of experts mentioned knowledge and accessibility of services and projects as most critical determinants of their acceptance and utilization. SP interventions should be designed to match the needs and characteristics of the target group, for example, in terms of content, language and imagery.

“The most successful interventions are those that start from the people’s needs […] and that are carried out with a vision that takes into account structural aspects and also established knowledge. And sometimes that lacks. Either they are bottom-up initiatives but not taking into account enough evidence and structural aspects. Or they are top-down measures which are very well thought sometimes but not rooted in the ground and not well prepared with regard to the inclusion and participation of the actors at several levels. We should try to find a middle way between bottom-up and top-down.” (Participant 24, female, science, Switzerland)

According to many interviewees, services and projects tailored to address the unique needs and characteristics of specific risk groups increase their acceptance and use. Low-threshold access is essential, encompassing aspects like online availability, anonymity and immediate availability. Services should cater to the different preferences of different target groups, such as the common preference for digital services among younger individuals or the preference for face-to-face contacts among older people.

“For these services, it is always relevant that they have low-threshold access, meaning low barriers. That means that it does not cost anything, that I can get help quickly and that I can also reach it easily in terms of physical distance.” (Participant 6, male, policy, Austria)

Many experts highlighted the value of including individuals with lived experience and their relatives in designing and implementing SP measures, thus enhancing their relevance, feasibility and effectiveness. Despite its acknowledged importance, actual involvement of these perspectives is often limited and insufficient in practice, according to most interviewees.

“As has been repeatedly highlighted by the World Health Organization, there is an ongoing demand for greater involvement of those affected in determining what is developed and subsequently implemented in suicide prevention. And, of course, another important group in this context is the relatives who have lost someone to suicide. This is certainly crucial. But there is still a significant need for catching up in this area as well.” (Participant 17, male, science, Austria)

The representation of affected individuals and relatives in SP associations was highlighted as a positive advancement.

“There is an umbrella organization of associations for those affected. And there are associations for those affected in almost every federal state. We collaborate very closely with them. In the SUPRA expert panel, two individuals from this umbrella organization and three relatives representatives are involved […] this is very important to us. It was missing in the beginning. For the first two or three years, no one was involved. Then we included them. And honestly, without that, it would not work at all.” (Participant 5, male, policy, Austria)

Several experts highlighted the importance of an encouraging, open and non-judgmental discourse on suicidality in both health care settings and the general population. Destigmatizing mental health issues and promoting personal, trusting relationships can substantially enhance participation in preventive measures. Furthermore, sharing first-hand experiences of overcoming personal crises and helpful coping strategies at public information events and in the media were described as a powerful tool for increasing acceptance and participation in SP services and projects.

“It is very important to destigmatize. […] that people get the feeling that it is okay and good and important that they get help when they are not well. That people talk about it. Public relations and media coverage are certainly important in this respect. […] With the Papageno effect, it is very impressive how important it is to include reports from individuals who have overcome suicidal crises themselves in the media and what a good role model effect that has.” (Participant 29, male, policy, Austria)

“In the realm of media, for example, we know that it is extremely effective when those who have experienced suicidal thoughts speak about their struggles and how they deal with them. Our evaluations show that this is much more effective than when experts, like you or me or someone else, talk about this topic.” (Participant 17, male, science, Austria)

#### 3.2.4 Further challenges, gaps and potentials for advancement in suicide prevention efforts

Providing adequate psychiatric and psychotherapeutic care to individuals with mental disorders is key to preventing suicides. However, several interviewees noted structural challenges within psychiatric and psychotherapeutic care, such as a shortage of specialists, long waiting times and the scarcity of suicide-specific therapeutic approaches. Experts from all three countries highlighted the necessity for strengthening psychiatric and psychotherapeutic care, including suicide risk assessment in these settings. In particular, care for individuals after a suicide attempt and support for relatives after a suicide should be enhanced.

In this context, disparities in mental health care between urban areas, including suburban agglomerations, and rural areas were highlighted. According to experts from all three countries, rural areas tend to have weaker health care coverage and stronger stigmatization of mental health problems. Access to mental health support is particularly difficult in cases of immobility and a lack of digital skills.

“In rural regions, the health care provision is still significantly poorer than in urban areas. This is why, in some rural regions, the suicide rates are indeed significantly higher than in cities. There is certainly a need for improvement.” (Participant 29, male, policy, Austria)

Some interviewees noted that lower suicide rates in urban areas are largely due to better psychosocial care and a stronger social safety net.

“Our areas of concern with the highest suicide rates are rural regions. We know which areas those are. And yes, it is true that suicide rates are generally lower in urban areas. This is quite noticeable. However, it does not necessarily correlate directly with the existing suicide prevention measures but rather with an overall better psychosocial care and a generally stronger social safety net.” (Participant 5, male, policy, Austria)

One of the most frequently mentioned gaps and potentials for optimization was the design and implementation of targeted SP programs and projects specifically designed for certain risk groups, such as men and older adults. Experts from all three countries pointed out that despite the considerably higher suicide rates observed in these two subpopulations, there are insufficient initiatives that aim to address their specific needs.

Furthermore, study participants from Germany and Austria emphasized the need to establish a national 24/7 telephone hotline that offers support in times of need and crisis.

#### 3.2.5 Impact of the COVID-19 pandemic on suicide prevention

Most interviewees from Germany, Austria and Switzerland reported a surge in demand for mental health services during the COVID-19 pandemic, particularly among children and adolescents. Many experts noted an increase in inquiries for mental health support and incidences of suicide attempts, especially among vulnerable groups, such as individuals with mental disorders.

“Now I am focusing once again on children and young people. There has been a massive increase in utilization [of clinical services]. And this applies throughout Switzerland. The inquiries, the registrations, especially with stress-associated problems – depression, eating disorders, compulsions, truancy – have gone up massively. And at the same time, the number of suicide attempts, from where we know it, has increased considerably.” (Participant 12, male, policy, Switzerland)

“Several child and adolescent psychiatry hospitals have seen an increase in suicidal behavior among their patients. […] already vulnerable children and adolescents were strongly affected and suffered a lot from the pandemic. […] These were vulnerable people, people who already had mental illnesses, people who worked in the health sector, people who were sitting at home in lockdown with school-aged children and were working from home. They had this double burden. These were basically our risk groups. And we saw that suicidality, depressiveness and such factors were higher [in these groups], as was anxiety.” (Participant 19, male, science, Austria)

According to several interviewees, individuals with pre-existing suicidal ideation seemed to experience the pandemic particularly stressful, which was reflected in more acute presentations of suicidality and an increased number of emergency admissions.

“We are counseling clients [adolescents and young adults] longer, more intensively. It feels like the clients come at a later stage in the crisis. They are already in a really bad state, that is really, really bad and not just bad or really bad. They come to us in a different state and then somehow need more and closer accompaniment. There was a lot of loneliness as an issue. And of course, the only way to prevent this is to get into contact and to maintain this contact for a while. Of course, we could not counsel more clients, because the capacity has remained the same. But the intensity has changed.” (Participant 14, female, practice, Germany)

Several experts from all three countries mentioned that the higher demand for psychiatric and psychotherapeutic support exceeded the health care system’s capacity. The existing challenges in mental health care, such as long waiting times and insufficient treatment resources, were further exacerbated by the pandemic. At the same time, measures for controlling COVID-19 transmission complicated care provision. In some facilities, for example, physical contact was temporarily restricted or even stopped completely.

“In the meantime, we know and it is also well documented that, for example, the number of young people who need help because they have fallen into depressive and suicidal crises has gone up quite a bit. And that the corresponding support services that are available are unfortunately not sufficient.” (Participant 31, female, practice, Austria)

Despite initial concerns and the perception of increased psychological distress, according to the interviewees, no significant increase in overall suicide rates was recorded during the first year of the COVID-19 pandemic in Germany, Austria and Switzerland. However, several experts cautioned about potential delayed effects on mental health and suicide rates, emphasizing the link between economic recessions and increased suicidality.

“What has not increased so far are suicides. Here, we need to focus very strongly on the recession. Because there is very good evidence that economic recessions with their effects, especially on the labor market, and here the keyword is unemployment, have an influence on suicides. […] We also know from other crises that the psychological, the mental health component always takes longer to heal. And when the medical aspects [of the pandemic] are no longer in the foreground, the danger that suicides will increase is actually greatest.” (Participant 17, male, science, Austria)

Besides the negative effects, several experts highlighted that the COVID-19 pandemic increased awareness of mental health and suicidality in society. This improvement was reflected, for example, in increased media coverage, public interest and political engagement in SP issues, leading to more support for mental health initiatives.

“I would say that to a certain extent, the COVID-19 pandemic has somehow played into our hands thematically. Things actually improved during the Corona period in that the topic of mental health and mental stress became more discussable. The stigma has, in my opinion, decreased to a certain extent. That is, because all people are burdened.” (Participant 5, male, policy, Austria)

“We definitely felt a stronger interest in the topic. Because before, generally the topic mental health was not discussed in parliament or in the media or anything like that. […] We have really received MANY parliamentary inquiries: what is the federal government doing to strengthen the population psychologically? […] It has given the issue a boost, also the topic of suicidality, from the population to the media and parliamentarians.” (Participant 9, female, policy, Switzerland)

According to the experts, the COVID-19 pandemic necessitated several adjustments to many SP measures. Interviewees reported a shift to telephone, video and online formats, for example in counseling centers. While face-to-face contact was temporarily restricted in all three countries, virtual events were introduced and in many cases broader audiences could be reached. Some public information events and research projects were put on hold. In other settings, services could be expanded. Furthermore, new projects and awareness campaigns emerged, some of which focusing on supporting mental health during the COVID-19 pandemic.

## 4 Discussion

An important finding of our study is that the experts’ statements on most topics did not differ considerably or systematically by country (Germany, Austria and Switzerland) or professional perspective (policy, science and practice). Overall, the interviewees identified similar hindering and enabling factors in SP measures and strategies.

This paper discusses our main findings within the broader context of existing scientific literature. Since experts have highlighted the value of national SP strategies in various contexts, we begin by examining the role of these initiatives. Following this, we discuss our findings on cross-sectoral and multi-stakeholder collaboration, as crucial components of an effective SP approach. We conclude with further specific recommendations to advance SP in Germany, Austria and Switzerland. Given the abundance of research dedicated to evaluating the impact of the COVID-19 pandemic on mental health, suicide rates and health care systems, we chose not to discuss our findings in this context.

### 4.1 Role of national suicide prevention strategies

According to SP experts from Germany, Austria and Switzerland, national SP strategies in these countries have been pivotal in increasing awareness and prioritizing SP efforts. Interviewees emphasized that these initiatives provide a structured framework to foster networking, collaboration and learning, while improving the visibility of SP issues and boosting political commitment to related efforts. This observation aligns with research indicating that political advocacy and policy change can help to address current and future public health issues and advancing public health initiatives (27). Furthermore, Platt et al. (5) confirm that national SP strategies improve the visibility of SP in policy agendas.

While scientific evidence on the effectiveness of individual SP interventions is available, reliable studies on the impact of national SP strategies as a whole are scarce (5). Due to factor such as their complexity and multi-component nature, the organic and non-linear development in strategy implementation, and difficulties to control for confounders and covariates, little is known about the effect of national SP strategies on suicide rates (5, 28). As reported by the WHO, some national strategies, for example in England, Scotland and Sweden, have contributed to reducing the suicide rate and increasing the awareness within society and among relevant stakeholders (29). According to Matsubayashi and Ueda (13) as well as Lewitzka et al. (28), national SP strategies can have a positive impact on reducing suicides. However, there are also studies that have reported no or even a negative effect of an implemented SP strategy (30–32). In addition to the many potential benefits of SP strategies and interventions, potential unanticipated negative consequences must be considered (33). Potential adverse events, such as increased maladaptive attitudes toward suicide and reduced help-seeking behavior, require thorough investigation to facilitate informed decision-making in SP programming and implementation (33).

Although national SP strategies usually have the overarching aim to reduce the suicide rate, we advocate that this outcome criterion alone is not sufficient. In addition to effectiveness and impact, criteria such as relevance, coherence, feasibility, adaptability and sustainability should be key indicators to evaluate the usefulness and successful implementation of an SP strategy (34). The International Association for Suicide Prevention highlight that in addition to the mere existence of a national strategy, its appropriate implementation and monitoring are important aspects that are lacking in many countries (30).

National SP strategies should be monitored and refined over time, informed by current scientific evidence on suicide characteristics and SP measures (30). Pirkis et al. (35) suggest that these initiatives could be enhanced by a stronger focus on the social determinants of suicidality. A whole-of-government approach, involving cross-sectoral collaboration and shared societal and governmental responsibility to SP as well as the involvement of individuals with lived experience, could address these determinants more effectively (35).

### 4.2 Cross-sectoral and multi-stakeholder collaboration in suicide prevention

According to the interviewees, there has been a positive trend toward enhanced interconnectedness and collaboration across various sectors and stakeholders pertinent to SP. Many experts attributed this improvement primarily to the implementation of national SP strategies and the increased awareness of mental health among stakeholders from, for example, politics, media and the health care sector. However, despite advancements, several experts indicated a persistent need for more robust collaboration at different levels. Existing regional efforts and networks should be leveraged as good practice models to strengthen collaboration among sectors, stakeholders and professions on a larger scale.

Previous research indicates that decisions in sectors outside the health system largely impact SP (27). Thus, cross-sectoral collaboration and action must be part of any SP strategy (27, 35). Whole-of-government approaches involve multiple government sectors such as health, media, criminal justice, transport, agriculture and education (12). Moreover, multi-stakeholder collaboration, which includes partnerships with non-governmental organizations and community stakeholders, is important for a comprehensive SP approach (12). Engaging stakeholders throughout the SP project and research cycle (co-ideation, co-design, co-implementation, and co-evaluation) can enhance SP outcomes (11). Cross-sectoral and multi-stakeholder collaboration facilitates the sharing of knowledge, best practices and lessons learned, provides opportunities for integrating SP into other initiatives and strengthens transparency and accountability among involved partners (12).

Despite progress in collaborative action, most national SP strategies and programs are predominantly developed, led and monitored by professionals anchored in the health sector (35). A more holistic approach, leveraging broad expertise from various sectors and stakeholders, could lead to more robust and coordinated SP efforts, thereby enhancing outcomes and elevating the societal prioritization of suicide (35).

To further streamline national and regional SP efforts, some experts highlighted the need for a central, national coordination center dedicated to SP. In Austria, such a coordination center was implemented in 2012 and is located at the national public health institute (36). Among other things, this institution is responsible for implementing SP measures and coordinating cross-sectoral and cross-regional efforts and collaboration (20). In Germany and Switzerland, the establishment of a central coordinating center is a promising avenue for advancing SP.

### 4.3 Further recommendations to advance suicide prevention in Germany, Austria and Switzerland

One of the most frequently mentioned gaps in SP efforts was the design and implementation of SP measures tailored to specific risk groups, particularly older people and men. In most developed countries, individuals aged 65 and older are considered ‘older adults’ (37), a group particularly vulnerable to mental disorders due to factors like limited mobility, chronic pain, social isolation and life-changing events like the loss of a spouse (38–40). In most countries worldwide, the suicide rate increases with age (1). However, research on old-age suicide and SP programs and projects for older adults are lacking (40, 41). In general, promoting healthy aging through supportive physical and social environments is crucial in preventing suicides in old age (38). Since many older individuals may favor personal, face-to-face interactions, providing in-person interventions should be prioritized (42). Furthermore, measures that address psychosocial adverse events (39) and training community members in identifying older individuals at suicide risk can contribute to prevent old-age suicides (43). Since many older individuals visit the primary care physician in the months preceding their suicide (44), training primary care providers to recognize suicidal thoughts is vital (39, 42). The currently scattered efforts and projects that aim to address old-age suicides should be comprehensively evaluated and, if proven effective, expanded to benefit more individuals.

The disparity in suicide rates between genders is striking, with men committing suicide more frequently than women. In 2019, the global age-standardized suicide rate for men was 12.6 per 100,000, compared to 5.4 per 100,000 for women (1). Such a pattern can also be observed in Germany, Austria and Switzerland, where the male-to-female suicide rate ratios are even higher (45–47). One possible reason for this disparity is that men are less likely to seek mental health support, for example, due to gender stereotypes and normative masculinities (48–50). Targeted SP measures tailored to men’s needs are scarce (10, 51). Related success factors include support from trusted individuals in informal settings, emotional regulation techniques and reframing help-seeking as masculine (51). Gender-sensitized approaches and peer-support can enhance the acceptance and effectiveness of SP measures (50, 52). Furthermore, strengthening social networks and destigmatizing mental illness are key in preventing suicides, especially among men (49, 50). Successful initiatives include, for example, an online SP campaign in Belgium (53), a peer-based SP campaign in Canada (54) and a multimodal workplace-based SP project in Australia (55). These measures have shown effectiveness in increasing help-seeking intentions among men with suicidal thoughts and fostering open discussions about mental health and suicidality. Again, such proven efforts should be scaled up and adapted in other regions and countries to reach more individuals in need.

Many experts emphasized the importance of tailoring SP measures to the specific characteristics and needs of the target group. In this context, they advocated for more substantial involvement of individuals with lived experience and their relatives in the design, implementation and evaluation of SP measures to enhance their relevance, feasibility and effectiveness. An increasing body of evidence supports the value of such engagement (11, 12, 35). This involvement can not only amplify the effectiveness but also bolster the sustainability of programs and projects (35). Beyond optimizing mental health care elements and the design and delivery of SP measures, individuals with lived experience should also have a greater role in SP policymaking (35).

Specific counseling services, for example in crisis intervention centers or via telephone, show promising results in contributing to decreasing suicide rates (56, 57). In this context, several experts from Germany and Austria highlighted the need for a national 24/7 telephone hotline for people in suicidal crises. Currently, both countries provide hotlines for counseling people with mental health problems, but these are either region-specific and/or are operated by religious organizations. Despite the fact that the services of religious organizations may offer denomination-independent counseling, an official, non-religious SP hotline would eventually be more widely accepted and utilized by the general population. Switzerland can be seen as a role model in this context. Two national hotlines, one for adults and another for children and adolescents, provide 24/7 free and anonymous psychosocial support to the general population in times of need and crisis. Both hotlines offer consultations in Switzerland’s three official languages (German, French, Italian) and partly also in English.

### 4.4 Limitations

Some limitations must be considered when interpreting our findings. This study presents the opinions, experiences and viewpoints of 36 individuals, which may not be generalizable to all SP experts in Germany, Austria and Switzerland. The selection of experts was based on the researchers’ judgment, and different experts might have highlighted alternative issues or brought different experiences to light. This condition is typical in qualitative research designs and limits the representativeness of our findings. We have carefully summarized the experts’ statements and included only the main results corroborated by a substantial proportion of the experts in our manuscript. Consequently, we assume that the likelihood of bias influenced by unfounded beliefs or emotions is minimal.

Given that the interviews were primarily conducted in German, with one interview in English, this represents a limitation, especially in the context of Switzerland’s multilingual landscape. Due to the federal structure of Switzerland, variations in SP activities and related experiences across different cantons should be anticipated. As a result, this research project does not adequately represent perspectives from French- and Italian-speaking cantons. Additionally, the viewpoints of individuals directly affected by suicidality and their relatives were not included, which would have provided deeper insights into the effectiveness, acceptance and key success factors of SP initiatives.

Investigating SP measures and strategies in Germany, Austria and Switzerland limits the applicability of many findings primarily to these countries and potentially others with comparable socio-cultural, economic and political-organizational characteristics. The findings might have varied if countries with different political systems, such as those featuring centralized planning and decision-making in public health, had been included in the analysis.

## 5 Conclusion

This qualitative study explores the challenges, gaps and success factors of SP in Germany, Austria and Switzerland, drawing on expert opinions. Although each country employs its own strategy, our study reveals many common threads in their SP landscape. The findings provide actionable guidance for advancing SP in Germany, Austria, Switzerland and potentially other countries with similar socio-cultural, economic and political-organizational characteristics. While considerable progress has been made, there remains a need to refine and strengthen collaborative and evidence-based SP efforts. The awareness and commitment of all relevant sectors and stakeholders are crucial in ensuring that SP is a prioritized, well-resourced and effective societal initiative.

## Data availability statement

The datasets presented in this article are not readily available because they contain information that could compromise the privacy of the interviewees. Requests to access parts of the datasets should be directed to the corresponding author (SW, sophia.werdin@swisstph.ch).

## Ethics statement

The studies involving humans were approved by the Ethics Committee of Northwestern and Central Switzerland (ID Req-2022-00881). The studies were conducted in accordance with the local legislation and institutional requirements. The participants provided their written informed consent to participate in this study.

## Author contributions

SW: Conceptualization, Data curation, Formal analysis, Investigation, Methodology, Project administration, Visualization, Writing – original draft, Writing – review & editing. KW: Conceptualization, Funding acquisition, Resources, Supervision, Writing – review & editing.
